# Impact of interpersonal relationships on academic burnout among trainee teachers: A comprehensive study

**DOI:** 10.1192/j.eurpsy.2024.387

**Published:** 2024-08-27

**Authors:** A. Bouhaba, Z. Boumaaize, Y. El Madhi, A. Soulaymani, A. Mokhtari, H. Hami

**Affiliations:** ^1^Laboratory of Biology and Health, Faculty of Sciences, Ibn Tofail University, Kenitra; ^2^Regional Center for Education and Training Professions, Rabat, Morocco

## Abstract

**Introduction:**

Recent research has identified varying levels of burnout among teachers, particularly those in training. This condition is believed to be influenced by a combination of internal factors, such as psychological characteristics, and external factors, such as work-related pressures and the social environment.

**Objectives:**

We examined the prevalence of burnout syndrome and assessed the potential risk factors associated with this condition. This study investigates the complicated correlation between academic burnout and interpersonal connections among trainee teachers in Morocco.

**Methods:**

We used a comprehensive database generated from the Maslach Burnout Inventory-Student Survey (MBI-SS), a questionnaire designed specifically for this study, and validated for this context. We examined various dimensions of academic burnout to unravel the complexity of this connection. Our study analyzed individual, professional, and social factors within a cohort of 732 prospective teachers in Morocco during the 2021/2022 academic year.

**Results:**

The findings revealed an intricate network of interrelated factors that contributed to the occurrence of academic burnout among trainee teachers. Significantly, the study highlighted the impact of interpersonal relationships on academic burnout. Trainee teachers who received support and positive interactions from colleagues and superiors showed significantly lower levels of academic burnout. Interpersonal relationships within the educational community also played a pivotal role in preventing burnout. Moreover, our multivariate analysis showed that certain sociodemographic factors,including age, gender, and prior educational experience, moderated the influence of interpersonal relationships on academic burnout.

**Conclusions:**

This study significantly contributes to the comprehension of academic burnout in trainee teachers by emphasizing the vital role of interpersonal relationships in this context. The findings emphasize the necessity of interventions that enhance interactions within educational institutions to prevent academic burnout and promote a healthy learning environment for trainee teachers.

**Disclosure of Interest:**

None Declared

